# Androgen Receptor Signaling in Prostate Cancer Genomic Subtypes

**DOI:** 10.3390/cancers13133272

**Published:** 2021-06-30

**Authors:** Lauren K. Jillson, Gabriel A. Yette, Teemu D. Laajala, Wayne D. Tilley, James C. Costello, Scott D. Cramer

**Affiliations:** 1 Department of Pharmacology, University of Colorado Anschutz Medical Campus, Aurora, CO 80045, USA; Lauren.Jillson@cuanschutz.edu (J.K.L.); Gabriel.Yette@cuanschutz.edu (G.A.Y.); Teelaa@utu.fi (T.D.L.); James.Costello@cuanschutz.edu (J.C.C.); 2 Department of Mathematics and Statistics, University of Turku, 20500 Turku, Finland; 3 Dame Roma Mitchell Cancer Research Laboratories, Adelaide Medical School, University of Adelaide, Adelaide, SA 5005, Australia; Wayne.tilley@adelaide.edu.au; 4 Freemason’s Foundation Centre for Men’s Health, University of Adelaide, Adelaide, SA 5005, Australia

**Keywords:** prostate cancer, Androgen receptor, AR, genomics, hormone receptor, lineage plasticity, therapy, resistance, subtype

## Abstract

**Simple Summary:**

Approximately 1 in 8 men will be diagnosed with prostate cancer (PCa) at some point in their lifetime. Five-year survival rate for patients with local or regionally spread disease is near 100%, but this drops to 30% when the cancer has spread to distant sites. Androgen receptor (AR) signaling plays a pivotal role in normal prostate development and PCa cell survival. Therapies targeting the AR pathway are mainline treatments, but resistance is a major clinical problem. From the literature and our own meta-analysis of PCa databases, we aimed to review the connections between PCa genetic alterations and the AR signaling axis at the primary stage. Assessing how combinations of PCa genetic drivers that arise in patients affect AR signaling will aid in stratifying patients who will likely respond to AR-directed therapies, and those who will require other therapeutic agents upfront in order to prevent disease progression.

**Abstract:**

While many prostate cancer (PCa) cases remain indolent and treatable, others are aggressive and progress to the metastatic stage where there are limited curative therapies. Androgen receptor (AR) signaling remains an important pathway for proliferative and survival programs in PCa, making disruption of AR signaling a viable therapy option. However, most patients develop resistance to AR-targeted therapies or inherently never respond. The field has turned to PCa genomics to aid in stratifying high risk patients, and to better understand the mechanisms driving aggressive PCa and therapy resistance. While alterations to the *AR* gene itself occur at later stages, genomic changes at the primary stage can affect the AR axis and impact response to AR-directed therapies. Here, we review common genomic alterations in primary PCa and their influence on AR function and activity. Through a meta-analysis of multiple independent primary PCa databases, we also identified subtypes of significantly co-occurring alterations and examined their combinatorial effects on the AR axis. Further, we discussed the subsequent implications for response to AR-targeted therapies and other treatments. We identified multiple primary PCa genomic subtypes, and given their differing effects on AR activity, patient tumor genetics may be an important stratifying factor for AR therapy resistance.

## 1. Introduction

Prostate cancer (PCa), the most commonly diagnosed cancer in men in the United States, remains the second leading cause of U.S. male cancer related deaths. The majority of cases (89%) are diagnosed when the disease is localized to the prostate and neighboring organs, providing a five-year survival rate of nearly 100%. However, once the disease has spread to distant sites, patients have a significantly lower five-year survival rate of 30% [1]. Developing novel therapies to treat metastatic PCa is critical. However, identifying tools to better stratify the large percentage of local disease cases and treat aggressive cases upfront is another important step in preventing PCa mortality.

The androgen receptor (AR), a steroid hormone nuclear receptor, is a critical transcriptional regulator of epithelial lineage programing in the prostate [2]. AR functions as a transcription factor activated by androgen ligands to control gene expression programs responsible for many functions, including cell differentiation, growth and survival [3]. The AR signaling axis is a critical dependence for many early and late stage PCa tumors, making AR-directed therapies a front-line treatment for local and metastatic disease [4,5,6]. These therapies include two general mechanisms of action: Depleting androgen hormone levels, known as androgen deprivation therapy [7], and directly inhibiting AR function using antiandrogens, such as enzalutamide. These therapies can delay disease progression by an average of 2–3 years, but most patients develop resistance with the emergence of castration-resistant PCa (CRPC) [8,9,10]. Unfortunately, at this point, there are not many other long-term CRPC therapies. The duration of response to ADT and antiandrogens, often in combination with other treatments, varies greatly among patients [11]. This poses the question of whether underlying molecular features of PCa differentially drive the response or lack thereof to these AR-targeted therapies. Despite many efforts, clinically validated biomarkers predicting response to ADT have yet to be identified [11,12].

Many genomic alterations drive PCa, most predominately fusions and copy number alterations, with mutations being less frequent [13,14,15,16]. The landscape of alterations becomes even more complex in metastatic disease, in part, due to varying selective pressures inflicted upon tumors at this stage [13,17]. Particularly after ADT, these selective pressures drive many changes in AR including *AR* gene amplification, rearrangements, mutations, and alternative splicing [10,18]. Alterations to the *AR* gene itself are largely restricted to the metastatic disease stage [15,19,20]. However, AR signaling, a central network in PCa, can still be altered at the primary stage. Many prevalent genomic alterations in primary PCa directly or indirectly influence AR signaling.

The development of better strategies to treat metastatic PCa is important and has been extensively covered in the literature. In this review, we will instead focus on genomic alterations that constitute primary PCa, and summarize the interplay between primary PCa genomics and AR. Studies evaluating primary PCa have largely been focused on individual genomic alterations. While these studies are important, single alterations largely do not confer a difference in survival or understanding of therapy resistance [15,21]. Most importantly, PCa, even at the early stage, is comprised of many co-occurring alterations. Mouse models repeatedly highlight the insufficiency of single drivers to promote disease development, and it is probable to assume the same is true in humans. In addition to discussing individual genetic alterations, we will highlight alterations that significantly co-occur and the joint implications these have on the AR axis. We will include a review of the current literature, as well as discuss our own meta-analysis of primary PCa databases. Finally, we will also review what is known about the relation of these genomic subtypes to AR-targeted therapy response and other therapies that have preclinical indications for specific alterations. 

## 2. Individual Alterations

**Figure 1:**
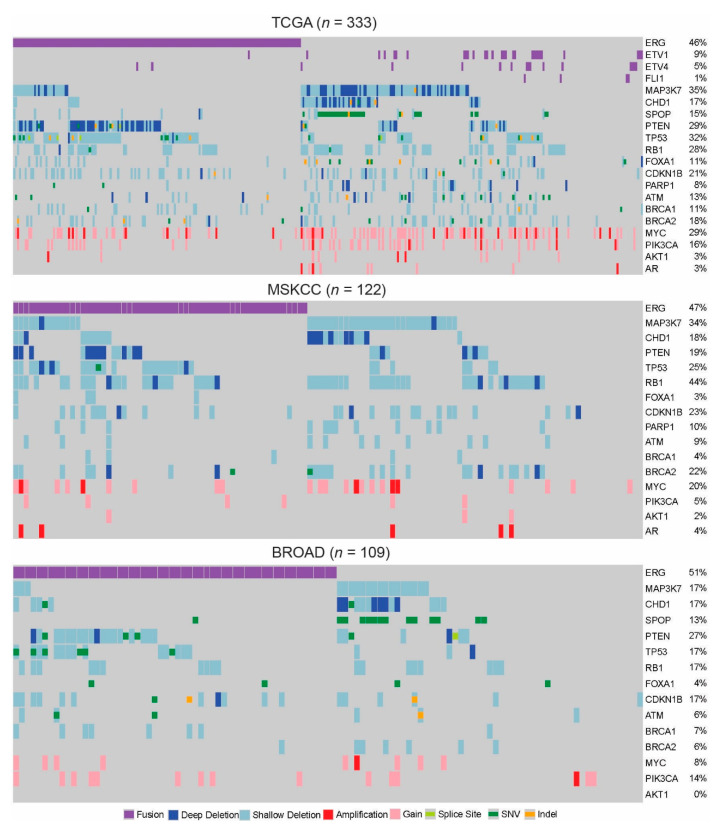
Genomic alterations across multiple primary PCa cohorts. Meta-analysis of three independent cohorts of primary PCa evaluating frequencies of the most common genomic alterations at this disease stage: TCGA [21], MSKCC [15], and BROAD [22]. The MSKCC dataset had few samples with mutation data, so mutational frequencies are underrepresented in this cohort. Shallow and deep deletions indicate heterozygous and homozygous deletions, respectively. Gain and amplification indicate low-level and high-level copy gain, respectively. Calls for alterations are based on algorithms used and parameters set within each cohort’s data repository.

**Figure 2:**
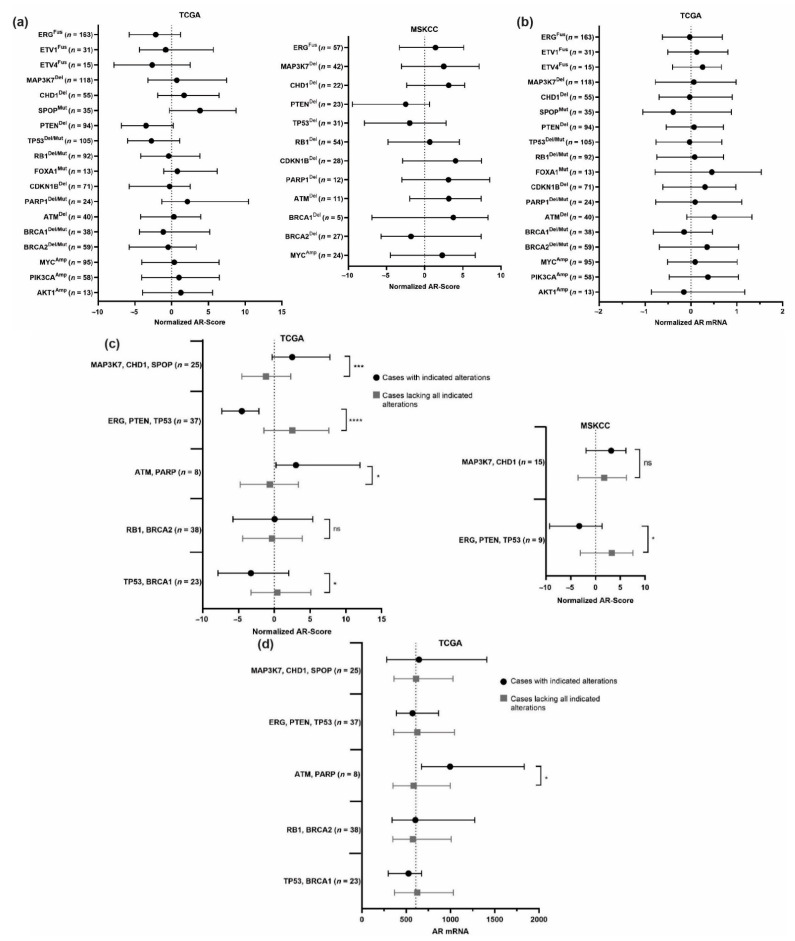
AR activity across primary PCa genomic subtypes. (**a**) Normalized AR-score values for patients with each indicated alteration in the TCGA and MSKCC cohorts. (**b**) Normalized AR mRNA reads for patients with each indicated alteration in the TCGA cohort. (**c**) AR Score values for subgroups of patients in the TCGA and MSKCC cohorts that contain the alterations listed versus patients that contain none of the alterations listed. (**d**) Normalized AR mRNA reads for subgroups of patients that contain the alterations listed versus patients that contain none of the alterations listed. All data points represent medians (point) with interquartile range (lines). The dotted line is the average across all patients in the cohort. Only available alterations or subgroups with at least 5 patients were included in analyses. Mann-Whitney test; * *p* < 0.01, *** *p* < 0.0001, **** *p* < 0.00001, ns = not significant.

In order to gain a comprehensive picture of the genomic landscape of primary PCa, we have evaluated the most common alterations across multiple independent cohorts: TCGA [21], MSKCC [15], and BROAD [22]. The distributions of these alterations within each cohort, based on the data available, are illustrated in Figure 1. While it is important to note that heterozygous and homozygous deletions can have different functional consequences, we have included both in our analysis to accurately represent the frequency of alterations to these genes. Based on available RNA expression data, we have evaluated trends for AR mRNA levels and AR-Score values across all individual alterations (Figure 2a,b). AR-Score is a generalized measure of AR transcriptional activity based on expression of a panel of validated AR target genes [23]. The AR-Score and *AR* mRNA levels are normalized across patients within each cohort. Evaluating *AR* mRNA does not completely reflect the AR protein level, which would be ideal for understanding the functional level of AR. Unfortunately, AR protein expression is not available for many cohorts, so *AR* mRNA will be used to generally assess potential changes to *AR* expression induced by PCa genomic alterations. AR-Score will be used as a better representation of downstream AR signaling activity.

Based on these analyses and the review of the literature, we will first discuss what is known about the mechanistic relationships between each individual alteration, the AR signaling axis, and AR-targeted therapies. However, as illustrated in Figure 1 and Table 1, there are alterations that significantly co-occur within patients. These “subtypes” are more clinically relevant to evaluate, so we will also discuss the joint implications of co-occurring alterations on the AR axis and treatment response.

### 2.1. ETS-Factors

The most common genomic alterations in PCa involve the fusions of ETS family transcription factors and AR responsive genes. The fusion between *ERG* and *TMPRSS2* is the most frequent, occurring in 46–51% of primary PCa cases (Figure 1) [24]. Other ETS family fusions including those of *ETV1*, *ETV4,* and *FLI1* occur less frequently in 1–9% of primary cases (Figure 1) [24,25,26]. ETS factor fusions in the prostate are early events and are not sufficient to drive PCa without concurrent alterations, such as *PTEN* deletions. However, the overexpression of ETS factors in mice can promote precancerous lesions and lead to increased invasion in human cultured cells [27,28,29,30]. Interestingly, AR plays a role in generating these fusion events by bringing these chromosomal regions in proximity [31,32,33].

*ERG* in particular exhibits significant crosstalk with AR, and these interactions along with the interplay with epigenetic modifiers have implications for PCa cell lineage programming. AR transcriptionally controls the differentiated state of prostate epithelial cells, while ERG functions to combat this and maintain a more de-differentiated cell state [34]. These two transcription factors control the divergent programs through a variety of actions. ERG and AR bind many of the same genes, such as *KLK3* (encoding PSA), but induce opposite changes in expression [35]. Additionally, ERG can directly control expression of AR and its target genes, as well as indirectly influence AR through interactions with HDAC1 [36,37,38]. Our meta-analysis in Figure 2a illustrates this inverse relationship, with *ERG* fusion positive cases exhibiting AR scores below or near the cohort average. This trend was also true for *ETV1* and *ETV4* fusions. In a small clinical study, *TMPRSS2:ERG* fusion tumors exhibited a potential increase in survival after ADT [39]. Alternatively, some preclinical models suggest that *ERG* fusion positive PCa may respond to high dose androgens [40]. Given that *ERG* fusions are very prevalent and often occur with many other alterations, its implication for therapeutic responses are likely context dependent. 

### 2.2. MAP3K7 

Deletions of *MAP3K7*, present in 17–35% of primary PCa cases (Figure 1), are some of the most common copy number alterations in primary PCa. *MAP3K7* is a member of the greater MAP kinase family that signals downstream to p38, JNK, and NF-kB, and is a bona fide tumor suppressor in PCa [41]. *MAP3K7* loss is sufficient for initiating precancerous and cancerous lesions in tissue recombination mouse models, is associated with high-grade PCa, and is linked to biochemical recurrence and prostatic brain metastases [42,43,44,45,46].

Despite the prevalence and clinical impact of *MAP3K7* loss, very few studies have evaluated its impact on AR activity. A putative mechanism of AR regulation by MAP kinases is direct phosphorylation, but a link between AR phosphorylation and *MAP3K7* loss has not been identified [47,48]. Our previous study demonstrated that *MAP3K7* loss, when co-lost with *CHD1*, is associated with increased AR activity [45]. Our meta-analysis supports these initial findings. Patients with *MAP3K7* deletions exhibited above average AR scores but not *AR* mRNA levels (Figure 2a,b). A previous study from our group also suggests that *MAP3K7* loss sensitizes PCa cells to DNA damage and cell cycle inhibitors through disruption of homologous recombination [49]. Many more studies focused on *MAP3K7* loss are needed however to better understand its mechanistic role in altering AR activity and promoting aggressive PCa.

### 2.3. CHD1

The deletion of *CHD1* is another common alteration in primary PCa. *CHD1* binds to promoters of actively transcribed genes marked with H3K4me3 and facilitates nucleosome turnover to activate transcription [50,51]. Present on chromosome 5q, *CHD1* is deleted in 17–18% of primary PCa (Figure 1). While *CHD1* loss is not sufficient alone for tumorigenesis in mouse models, it is a PCa tumor suppressor and cooperates with other genomic alterations to drive disease progression [42,45,52]. While no direct interaction has been identified between AR and CHD1, the CHD1 interactome does involve nuclear receptor cofactors [52]. Recent studies have discovered that loss of *CHD1* affects the distribution of AR chromatin binding. Many loci are differentially or newly bound by AR, resulting in more oncogenic expression profiles [45,52]. In concordance with its effects on AR chromatin distribution, patients with *CHD1* loss exhibited higher than average AR scores, but not *AR* mRNA levels in our meta-analysis (Figure 2a,b).

The loss of *CHD1* has also been shown to promote AR-directed therapy resistance via dysregulation of chromatin [53]. This dysregulation permits more lineage plasticity, allowing other transcription factors to drive PCa cells away from the traditional luminal, AR-dependent lineage. Interestingly, *CHD1* expression was specifically associated with resistance to AR antagonist therapies but not to abiraterone treatments, an androgen synthesis inhibitor. While this study involved a small clinical cohort, it still might suggest that patients with loss of *CHD1* would benefit more from androgen withdrawal treatment [53]. Our recent study demonstrates that *CHD1* loss might contribute to enzalutamide resistance at the primary disease stage, as well and predict biochemical recurrence [45]. Loss of *CHD1* has also been associated with sensitivity to other potential therapies including DNA damaging agents [54,55].

### 2.4. SPOP 

The most frequently mutated gene in PCa is *SPOP*, occurring in 13–15% of primary PCa cases (Figure 1). SPOP functions as an E3 ubiquitin ligase adaptor protein, and confers the substrate specificity for the ubiquitination complex [56]. Mutations in *SPOP* often result in the loss of binding and subsequent increased expression of its targets [57,58,59]. The expression of the common *SPOP* F133 mutant is capable of enhancing the invasiveness of PCa cells and driving prostate tumorigenesis in vivo in the context of other alterations [56,60].

AR and many AR cofactors are well-characterized SPOP substrates in PCa [56,60]. Dysregulation of these by mutant SPOP contributes to increased AR protein and AR target gene expression [21,61,62,63]. Consistent with these findings, increased AR activity is seen in PCa cell lines and tumors with *SPOP* mutations [64]. In the TCGA primary PCa cohort, *SPOP* mutations had higher AR transcriptional activity than any other alteration [21,60]. Our meta-analysis highlights similar patterns. Patients with *SPOP* mutations exhibited AR-scores significantly higher than average (Figure 2a). Due to the positive effects on AR protein levels and activity, it is likely that tumors with *SPOP* mutations would be more susceptible to androgen deprivation or AR-targeted therapy. While more work needs to be done to make this association in patients, PCa cells expressing mutant SPOP are more sensitive to androgen deprivation [40]. *SPOP* mutations may also confer sensitivity to an HDAC3 inhibitor due to its upstream regulation of AR, as well as resistance to BET inhibitors due to the stabilization of the SPOP substrate BRD4 [65,66].

### 2.5. PI3K Pathway

The Phosphoinositide 3-kinase (PI3K) pathway is often altered in primary PCa through genetic alterations in components such as *PTEN, PIK3CA,* and *AKT1*. *PTEN* is an important negative regulator of the pathway and at least one copy is deleted in 19–29% of primary PCa (Figure 1) [67]. Loss of *PTEN* generally leads to increased AKT activity and is associated with poor outcome [68,69]. Although less frequent, other members of this pathway including *PIK3CA* and *AKT1* are mutated or amplified in 5–16% and 2–3% of cases, respectively (Figure 1).

Many studies have shown an inverse relationship between AR signaling and PI3K pathway activation [70,71,72,73]. AR target gene, *FKBP5*, stabilizes PHLPP1 and PHLPP2, important phosphatases in the pathway, allowing for deactivation of phospho-AKT. This feedback loop can, in turn, contribute to AKT-driven growth in castrate conditions, as androgen deprivation leads to decreased FKBP5, PHLPP1 and PHLPP2 and increased AKT activation. AKT is also able to phosphorylate AR and mark it for ubiquitination and degradation [74]. Our meta-analysis of primary PCa highlights the negative association between the PI3K pathway and AR. Cases with *PTEN* loss displayed significantly below average AR scores in multiple cohorts (Figure 2a). Interestingly, cases with *PIK3CA* amplifications demonstrate slightly above average AR scores and *AR* mRNA levels, but it is hard to differentiate its independent effects and those of co-occurring alterations (Figure 2a,b). Success of PI3K pathway inhibitors has been limited in clinical trials, and given the reciprocal relationship with AR, targeting one or the other independently would likely lead to resistance [75,76]. Further studies evaluating the potential dual inhibition of the PI3K and AR pathways are warranted to better understand the potential clinical value in this approach. 

### 2.6. TP53 

*TP53* participates in many tumor suppressing functions, and it is deleted or mutated in 17–32% of primary PCa (Figure 1). This frequency greatly increases in metastatic CRPC disease where *TP53* loss is present in over 50% of cases [13]. Genetic mouse models demonstrate insufficiency of *Trp53* loss to drive disease progression on its own, although some studies have seen evidence of precancerous events [77,78]. Given its role in DNA damage response, the loss of *TP53* also permits accumulation of more genomic changes that can contribute to PCa disease progression [79,80].

Studies suggest that p53 intricately contributes to the regulation of AR. In PCa cell lines, inhibition of p53 or *TP53* overexpression both decreased AR-mediated signaling, and *TP53* loss of function was sufficient to decrease AR protein expression [81]. These results suggest that a tight balance in p53 and AR levels is important for normal AR function, and that disruption to this may contribute to disease progression. Likely, the clinical effects of *TP53* gene alterations on AR activity are dependent on other co-occurring alterations. In our analysis, cases with *TP53* deletions or mutations exhibited below average AR scores and average *AR* mRNA levels across multiple cohorts, indicating that loss of *TP53* in primary PCa generally leads to decreased AR activity (Figure 2a,b). *TP53* expression may also have implications for sensitivity to chemotherapy in PCa. PCa cell lines transfected with a dominant negative p53 mutant showed decreased sensitivity to chemotherapy, while transfection of more advanced PCa cells lines that have mutant *TP53* with *WT-TP53* increased sensitivity [82].

### 2.7. Cell Cycle: RB1 and CDKN1B

Retinoblastoma, *RB1*, exhibits almost exclusively heterozygous deletions at the primary stage, occurring in about 17–44% of cases (Figure 1). Mouse studies have shown that a loss of *Rb1* alone causes hyperplasia that slowly progresses to PIN formation and concomitant increase in proliferation [78,83]. Other than its control over cell cycle progression, RB1 also plays a role in regulating AR activity. *RB1* loss increases E2F-1 activity and expression of many of its target genes, including AR [83,84]. Subsequently, *RB1* loss results in increased AR target gene expression and castrate resistant growth in PCa cell lines. In our meta-analysis of primary PCa, but the loss of *RB1* displays average AR scores and *AR* mRNA levels across cohorts (Figure 2a,b).

*CDKN1B* is another component of cell cycle regulation that is altered in PCa. An important inhibitor of G1-S cell cycle progression, *CDKN1B* is lost or mutated in 17–23% of primary PCa (Figure 1). *CDKN1B* alterations have not been widely studied in PCa, but deletions of *CDKN1B* have recently been associated with development of metastasis in African American men with clinically localized PCa [85]. These are initial findings in a small clinical study, however, and need to be verified in other cohorts. Additionally, not much is known about the effects of *CDKN1B* loss on AR. Our meta-analysis shows an association between *CDKN1B* deletions, average to above averge AR scores, and slightly elevated *AR* mRNA levels (Figure 2a,b). However, *CDKN1B* deletions significantly co-occur with alterations such as *MAP3K7* and *CHD1* deletions that increase AR activity so the exact role of *CDKN1B* in AR regulation is hard to decipher (Table 1).

### 2.8. FOXA1

*FOXA1*, mutated or lost in 3–11% of primary PCa, encodes a pioneer factor that binds closed chromatin and facilitates chromatin accessibility for transcription factors (Figure 1) [86,87]. The majority of *FOXA1* coding sequence mutations are located in the DNA binding domain, and they can either decrease FOXA1 DNA binding affinity or increase its activity and nuclear movement [21,88,89]. These particular DNA binding domain mutations are not enriched in metastatic disease, and thus, are thought to originate at the local stage [88].

The role of FOXA1 in the promotion of prostate cell proliferation is largely attributable to its interaction with AR as its pioneer factor. FOXA1 directly binds AR via interactions between their DNA binding domains, and it is important for directing prostate lineage specific AR programs and restricting non prostatic AR bindings events [90,91]. Mutations in *FOXA1* can subsequently alter these AR-directed programs in PCa. However, the effects that *FOXA1* mutations have on AR signaling seem to be dependent on the specific mutations present and the models used to study them. The results have shown both enhanced AR transcriptional activity in patient tumors and mouse prostate organoids, as well as downregulation of AR signaling [21,88,89,92,93]. Some mutations that decrease FOXA1 DNA binding ability result in stronger AR binding, sequestering it away from DNA [89]. Alternatively, increased expression of *FOXA1* or the presence of activating *FOXA1* mutations demonstrate more active AR that is responsive to lower levels of DHT [94]. In our meta-analysis, patients with *FOXA1* mutations displayed slightly above average AR scores and *AR* mRNA levels (Figure 2a,b). Due to the divergent effects of various *FOXA1* mutations, it is challenging to understand how *FOXA1* mutations broadly affect response to AR-targeted therapies. More in-depth studies on specific *FOXA1* mutants in both preclinical models and patient samples are needed to better understand their clinical impact.

### 2.9. DNA Repair: ATM, PARP1, BRCA1, BRCA2 

Many genes involved in DNA repair are altered in PCa, and this in turn contributes to the genomic instability and array of genomic alterations present in PCa. *ATM* (6–13%)*, PARP1* (8–10%)*, BRCA1* (4–11%) and *BRCA2* (6–22%) are some of the most common DNA repair genes deleted or mutated in primary PCa (Figure 1). Changes to the DNA damage response (DDR) pathway through these genetic alterations significantly contribute to PCa disease progression. The loss of function of homologous recombination (HR) genes, such as *BRCA1/2* can result in greater dependency on error-prone non-homologous end joining (NHEJ), consequently increasing the mutational burden in PCa [95]. The loss of *PARP1* and *ATM* also contribute to the genomic instability of PCa through their critical roles in recruiting repair proteins and coordinating repair with cell cycle arrest, respectively [96,97].

AR and DNA repair pathways contribute to each other’s regulation, which has interesting implications for treatment resistance. DNA damage causes an increase in AR activity to promote cell survival and induce expression of DNA repair genes [98]. PARP1 specifically is recruited to AR-bound sites and promotes AR transcriptional activity [99]. Inhibition of AR therefore reduces the function of DNA repair, particularly NHEJ [100]. Based on the role of PARP1 in promoting AR transcriptional activity, we might expect *PARP1* loss to decrease AR activity in patients. While *PARP1* deletions are associated with average *AR* mRNA levels, they are consistently associated with above average AR scores in multiple cohorts (Figure 2a,b). The broader effects of *PARP1* loss on DNA repair activity may instead contribute to the subsequent increase in AR activity. Interestingly, patients with *ATM* deletions also showed an association with above average AR scores and *AR* mRNA levels (Figure 2a,b). *BRCA1/2* alterations showed differing associations across cohorts, likely due to differences in co-occurring alteration frequencies (Figure 2a,b). However, larger cohorts are needed to make significant associations between these DNA repair genes and AR activity.

The interplay between AR and DNA repair is important for therapies as inhibition of one pathway may be mitigated by activity of the other. For example, having increased DDR activity in PCa with intact AR signaling can contribute to radiotherapy resistance [100]. This relationship between AR and DNA repair has also opened the door for the combination therapy involving ADT and radiotherapy [101,102].

### 2.10. MYC

One of the more commonly amplified regions in primary PCa is 8q24 which includes the *MYC* gene locus [103]. Amplification of this region is associated with *MYC* expression, and *MYC* amplifications are present in 8–29% of primary PCa (Figure 1) [104]. MYC is a transcription factor that regulates many cellular processes, including ribosome biogenesis and metabolism in PCa [105,106]. *MYC* overexpression was sufficient for induction of PCa in a genetic mouse model, and in humans *MYC* amplification occurs early in disease development and is predictive of recurrence after radical prostatectomy [104,107,108,109]. 

In both normal prostate tissue and PCa, AR and MYC exhibit a negative relationship. AR signaling and treatment with androgens negatively regulates *MYC* expression, and *MYC* overexpression downregulates many androgen response pathways [110,111]. While *MYC* expression is important in general for PCa cells, *MYC* overexpression only confers a growth advantage in the absence of androgens [111,112]. Additionally, *MYC* overexpression is sufficient to increase growth of androgen-dependent PCa cell lines treated with AR antagonists, and in CRPC is associated with AR-v7 levels [111,113]. In our meta-analysis of primary PCa databases, patients with *MYC* amplification exhibit roughly average AR scores and *AR* mRNA levels based on cohort distributions (Figure 2a,b). Given that the effects of *MYC* amplification on growth are largely compensatory in the absence of androgens, the lack of impact on *AR* levels and activity is not surprising. These findings suggest that *MYC* overexpression may contribute to resistance to AR-targeted therapies through the promotion of compensatory growth pathways under androgen depleted conditions. Because of its important role in PCa, there are ongoing efforts to target MYC directly and indirectly for therapeutic intervention [114,115].

## 3. Genomic Subtypes

While it is valuable to mechanistically study individual genomic alterations, it is more clinically relevant to understand the impact of multiple alterations that often occur together. Other studies have delineated “subtypes” of primary PCa based on multiple factors. However, these are largely centered on single alterations, as observed in the seven subtypes highlighted in the TCGA study [21]. Instead, we will define subtypes as groups of significantly co-occurring alterations (Table 1). We have identified five genomic subtypes of primary PCa that each involve at least two significantly co-occurring alterations: *MAP3K7/CHD1/SPOP; ERG/PTEN/TP53; ATM/PARP1; BRCA1/TP53; BRCA2/RB1.* These subtypes are highlighted in Table 1, along with mutual exclusivity of alterations between subtypes, and other alterations that co-occur broadly with aspects of multiple subtypes.

Given the centrality of the AR axis to PCa development, progression, and treatment resistance, the impact that genetic alterations have on AR may contribute to the mechanisms permitting co-occurring alterations and restricting mutually exclusive ones. We have evaluated the impact of our defined subtypes on AR Score (Figure 2c) and *AR* mRNA levels (Figure 2d) across the multiple cohorts. Based on the literature and our own analysis we will review what is known about their joint implications on AR activity and related therapies.

### 3.1. MAP3K7, CHD1, SPOP

One significant subtype present in primary PCa includes *CHD1* and *MAP3K7* deletions. Deletions of *CHD1* rarely occur without *MAP3K7* loss (Figure 1, Table 1), and the co-loss of *CHD1* and *MAP3K7* is largely mutually exclusive from *ERG* fusions and *PTEN* deletions. Our group has evaluated the implications of *MAP3K7* and *CHD1* co-loss on the AR axis and have found that PCa cells with this subtype exhibit increased AR activity, resistance to enzalutamide, and increased AR splice variant levels. Additionally, patients with decreased expression of these genes represent an aggressive subset who are more likely to relapse after radical prostatectomy [45].

Based on our analysis, *SPOP* mutations also significantly co-occur with *MAP3K7* and *CHD1* deletions (Figure 1, Table 1). The overlap of *SPOP* mutations and *CHD1* deletions has been of interest in the field in recent years. In a cohort of mCRPC, *SPOP* mutations and *CHD1* deletions were associated with abiraterone sensitivity marked by longer time on treatment and higher response rates [116]. Studies spanning all three genes individually and in pairs have also shown a synergistic sensitivity to DNA damage with potential response to PARP inhibitors or DNA damaging agents [7,49]. Based on the independent roles that *SPOP* mutations, *CHD1* deletions, and *MAP3K7* deletions have on increasing AR activity, patients with all three alterations are likely to have striking enhancements to AR signaling. In our meta-analysis, patients with all three alterations do, in fact, have higher than average AR scores and represent a subtype with some of the highest AR activity (Figure 2c). Based on these findings, it would be interesting to explore the potential of androgen deprivation therapy along with DNA damaging agents in models of this subtype. Further studies would be needed to understand how patients with this subtype respond to these therapies.

### 3.2. Alterations Mutually Exclusive from ERG/ETS Fusions

*ERG* fusions, which are the most common alterations in primary PCa, constitute a major component of other subtypes. As mentioned above, *ERG* fusions are mutually exclusive from *MAP3K7* and *CHD1* deletions. Additionally, multiple genomic analyses of primary PCa including our analysis in Table 1, show that *SPOP* mutations and *ERG/ETS* fusions are mutually exclusive [21]. This may be due to competing divergent transcriptomic changes between *SPOP* mutant induced AR signaling and *ERG* fusion signaling [40]. *SPOP* mutations are also mutually exclusive from *PTEN*, *TP53* and *BRCA1* alterations, all of which frequently occur with *ERG* fusions (Table 1).

*ERG* fusions have been linked to the activation and overexpression of *MYC,* leading to the repression of epithelial differentiation genes [34]. This could explain the low frequency of *ERG* fusion positive tumors with *MYC* amplifications, since *ERG* fusions are sufficient for driving increased *MYC* expression and activity. Our analysis also found that *PARP1* alterations and *ERG* fusions are mutually exclusive. This perhaps is because PARP1 and ERG interact, and PARP1 is required for ERG-mediated transcription [117]. Taken with PARP1 involvement in regulating AR transcriptional activity, the loss of *PARP1* in *ERG* fusion positive cancers would disrupt *ERG* expression and transcriptional activity [118].

*MYC*, *FOXA1*, and *PIK3CA* alterations are all mutually exclusive from *ERG* fusions and tend to co-occur with alterations spanning multiple subtypes we discuss (Figure 1, Table 1). These trends could indicate that these are early events in the development of PCa, or that alterations to these genes are advantageous in many situations. Given the complex interplay that each of these alterations has with the AR axis, the impact on AR may be contextually driven by specific co-occurring alterations.

### 3.3. ERG, PTEN, TP53

Given the prevalence of *ERG* fusions, PCa has often been delineated as *ERG* fusion positive or negative. While *ERG* fusions are mutually exclusive from the alterations described above, they significantly co-occur with many others, including *PTEN* deletions, *TP53* alterations, *RB1* deletions. Many studies have evaluated the impact that *ERG* fusions and *PTEN* deletions have on PCa tumorigenesis together, leading to increased tumor size and invasiveness [119,120,121]. Individually, *ERG* fusions and *PTEN* deletions exhibit an association with decreased AR scores in our meta-analysis (Figure 2a). However, in the context of their co-occurrence, studies have discovered a different impact on the AR axis. In a genetic mouse model with *PTEN* homozygous loss, *ERG* fusion restored AR activity, increasing AR binding and transcriptional programming [121]. In the setting of intact *PTEN* however, *ERG* fusions can prevent and restrict the luminal differentiation program controlled by AR. Upon *PTEN* loss, the role of these fusions seems to support some AR activity to cooperate with oncogenic PI3K pathway activity [121]. Additionally, a preclinical analysis by one group suggests that in the setting of *PTEN* loss, *ERG* expression maintains some AR transcriptional programming to promote resistance to AKT and AR dual inhibition [122].

In therapy resistant prostate tumors, the combination of *PTEN* deletions and *TP53* alterations has been associated with lineage plasticity involving decreased expression of *AR* and luminal differentiation genes. These tumors were more resistant to antiandrogen therapy, which is likely due to the lineage plasticity phenotype [123]. However, these two alterations also significantly occur with *ERG* fusions, and it is important to study the complete clinical context. Interestingly, another group found that the addition of *ERG* expression prevented the decrease in *AR* and luminal differentiation gene expression seen with *PTEN/TP53* deficiencies alone [124]. Instead, through regulation of cell-cycle genes and RB1 activity, *ERG* repressed E2F1-mediated mesenchymal gene expression and maintained AR activity. Whereas, *TP53/PTEN* deficient tumors resisted antiandrogen therapy, the addition of *ERG* fusions sensitized these to antiandrogen therapy. Alternatively, in *ERG* negative mouse tumors with *Trp53/PTEN* losses, CDK4/6 inhibitors were more successful in inhibiting tumor growth [124]. When comparing patients in our PCa cohort analysis with all three alterations to those lacking all three, the co-occurring group exhibited significantly lower AR scores despite what the literature might suggest (Figure 2c). It is important to note, however, that AR score is just one measure of AR activity and does not capture all changes to AR signaling. Overall, these findings suggest differential therapy indications for *TP53/PTEN* deficient tumors with or without *ERG* fusions, but more studies are needed to understand if these therapeutic strategies translate to patients with these subtypes. 

### 3.4. ATM and PARP1 

PARP inhibitors have more recently emerged as treatments for PCa with alterations in HR genes such as *BRCA1* and *BRCA2* due to the dependency of these tumors on PARP function [125,126]. This synthetic lethal treatment causes vast accumulation of DNA damage resulting in cell death. Interestingly, in our analysis we found that deletions of *PARP1* and *ATM* significantly co-occur (Figure 1, Table 1). This suggests that the synthetic lethality of targeting two aspects of the DDR pathways does not apply to all DDR genes. This is reflected in the decreased efficacy of PARP inhibitors in treating *ATM* deficient CRPC [127,128,129,130]. In our individual alteration meta-analysis, *PARP1* deletions were associated with an increased AR score, and *ATM* deletions were associated with increased *AR* mRNA levels. Patients with dual loss of these genes also exhibit significantly higher AR scores and *AR* mRNA levels than those without these alterations (Figure 2c,d). Given that dual loss of these two genes likely results in an increase in DNA damage, AR signaling may be stimulated to promote cell survival. Fortunately, PARP inhibition in combination with ATR inhibition has shown promise [129,131,132]. However, the combination of ADT may be important in this setting to prevent AR-driven cell survival. 

### 3.5. BRCA1/TP53 and BRCA2/RB1

*BRCA1* and *BRCA2* deletions or mutations are largely mutually exclusive from each other and tend to co-occur with different subsets of alterations. *BRCA1* significantly occurs with *TP53* alterations while *BRCA2* more commonly occurs with *RB1* loss, *PTEN* loss, or *MYC* amplifications (Figure 1, Table 1). Co-loss of *RB1* and *BRCA2* has been evaluated in PCa and is often due to large chromosomal deletions on chromosome 13. PCa cell lines and patient derived mCRPC organoids with *RB1* loss and *BRCA2* mutations demonstrated resistance to ADT and showed enrichment of EMT and invasion pathways [133]. In our analysis, patients with co-loss of *RB1* and *BRCA2* demonstrate average AR scores and *AR* mRNA levels that are not significantly different from patients without these alterations (Figure 2c,d). This suggests that this subtype may not have a significant impact on the AR signaling axis, at least not at the primary stage with largely heterozygous deletions of *RB1*. Patients with alterations in *TP53* and *BRCA1,* but do exhibit significantly decreased AR scores, most likely driven by the loss of *TP53* (Figure 2c). While PARP inhibitors have shown success for *BRCA1/2* deficient tumors, the effect of co-occurring alterations on treatment response is largely unknown. Current studies are investigating combination therapies including the addition of PI3K/Akt inhibitors to PARP inhibitors for HR deficient tumors, which may be important for *BRCA2* deficient PCa tumors that exhibit *PTEN* co-loss [126].

## 4. Conclusions

Here, we reviewed how common genomic alterations in PCa, and significant subtypes of co-occurring alterations, impact the AR signaling axis. We combined what is known mechanistically from the literature, as well as analysis of AR activity measures from PCa patient cohorts, in order to examine trends of AR activity across these subtypes. In general, genomic alterations either increase AR activity to facilitate survival, decrease AR activity and allow for lineage plasticity, or contribute to genomic instability that may further impact androgen independence. We noted that significantly co-occurring alterations typically have similar effects on AR activity. Whereas, mutually exclusive alterations have opposing effects. This suggests that in general, tumors must decide between maintaining AR activity or becoming more plastic. This could have valuable clinical implications in developing therapies that stimulate synthetic lethality.

While we focused on potential patient response to the widely prescribed ADT and AR antagonist therapies, it is important to note that other AR directed therapies are being evaluated for PCa. Supraphysiological androgen therapy and selective androgen receptor modulators (SARMs) are promising therapeutic strategies under investigation [134,135,136]. It will be interesting to see if these treatments can combat genomic subtype driven, oncogenic AR activity and re-establish a growth-inhibitory, differentiated AR program. 

There are however a few caveats to our own analysis, as well as significant hurdles when it comes to the application of tumor genomics in the clinic. The currently available patient data for PCa, including the cohorts used in this review, largely involve bulk tumor RNA-Seq. This along with tumor heterogeneity limits some of the interpretations that can be made about genomic alterations in patients. It is unclear if some of the co-occurring alterations occur in the same cells, mosaically within the same tumors, or represent independent multifocal areas of disease. The multifocal and heterogeneous nature of PCa poses challenges for applying tumor genomic analysis to inform treatments. The growing application of single cell -omics analysis will hopefully aid in elucidating the nature of co-occurring alterations, as well as their effects on the AR axis. With continued advancements in the field and more clinical studies, tumor genetics could be used to inform treatment decisions. This may help delineate patients who will likely do well on AR monotherapies, be inherently resistant to AR therapies, or need treatment combinations to combat the inevitable acquired resistance to AR single agent therapies.

## Figures and Tables

**Table 1:** Co-occurrence and mutual exclusivity of alterations in the TCGA cohort.

Subtype	A	B	Neither	A Not B	B Not A	Both	Log2 Odds Ratio	*p*-Value	*q*-Value	Tendency
*MAP3K7*, *CHD1*, *SPOP*	*MAP3K7*: HOMDEL HETLOSS	*CHD1:* HOMDEL HETLOSS	203	76	12	42	>3	<0.001	<0.001	Co-occurrence
	*MAP3K7*: HOMDEL HETLOSS	*SPOP*: MUT = MISSENSE	210	88	5	30	>3	<0.001	<0.001	Co-occurrence
	*CHD1*: HOMDEL HETLOSS	*SPOP*: MUT = MISSENSE	272	26	7	28	>3	<0.001	<0.001	Co-occurrence
	*CDKN1B*: HOMDEL HETLOSS	*MAP3K7*: HOMDEL HETLOSS	180	35	83	35	1.117	0.004	0.017	Co-occurrence
	*CHD1*: HOMDEL HETLOSS	*PARP1*: HOMDEL HETLOSS	264	45	15	9	1.816	0.007	0.033	Co-occurrence
	*MAP3K7*: HOMDEL HETLOSS	*PARP1*: HOMDEL HETLOSS	209	100	6	18	2.648	<0.001	<0.001	Co-occurrence
Exclusive from *ERG*	*ERG*: FUSION	*MAP3K7*: HOMDEL HETLOSS	92	123	89	29	−2.037	<0.001	<0.001	Mutual exclusivity
	*ERG*: FUSION	*ETV1*: FUSION	153	151	28	1	<−3	<0.001	<0.001	Mutual exclusivity
	*ERG*: FUSION	*CHD1*: HOMDEL HETLOSS	135	144	46	8	−2.617	<0.001	<0.001	Mutual exclusivity
	*ERG*: FUSION	*PARP1*: HOMDEL HETLOSS	158	151	23	1	<−3	<0.001	<0.001	Mutual exclusivity
	*ERG*: FUSION	*FOXA1*: MUT	168	152	13	0	<−3	<0.001	0.002	Mutual exclusivity
	*ERG*: FUSION	*MYC*: AMP GAIN MUT = MISSENSE	115	123	66	29	−1.283	<0.001	0.002	Mutual exclusivity
	*ERG*: FUSION	*ETV4*: FUSION	167	150	14	2	−2.652	0.005	0.022	Mutual exclusivity
	*ERG*: FUSION	*SPOP*: MUT = MISSENSE	146	152	35	0	<−3	<0.001	<0.001	Mutual exclusivity
Exclusive from *SPOP*	*TP53*: HOMDEL HETLOSS MUT	*SPOP*: MUT = MISSENSE	194	104	34	1	<−3	<0.001	<0.001	Mutual exclusivity
	*PTEN*: HOMDEL HETLOSS	*SPOP*: MUT = MISSENSE	207	91	33	2	−2.859	<0.001	0.004	Mutual exclusivity
	*SPOP*: MUT = MISSENSE	*BRCA1*: HOMDEL HETLOSS MUT	260	35	38	0	<−3	0.011	0.045	Mutual exclusivity
*ERG*, *PTEN*, *TP53*	*ERG*: FUSION	*PTEN*: HOMDEL HETLOSS	151	89	30	63	1.833	<0.001	<0.001	Co-occurrence
	*PTEN*: HOMDEL HETLOSS	*TP53*: HOMDEL HETLOSS MUT	181	47	59	46	1.586	<0.001	<0.001	Co-occurrence
	*ERG*: FUSION	*TP53*: HOMDEL HETLOSS MUT	141	87	40	65	1.397	<0.001	<0.001	Co-occurrence
	*PTEN*: HOMDEL HETLOSS	*BRCA2*: HOMDEL HETLOSS MUT	206	68	34	25	1.155	0.006	0.028	Co-occurrence
*ATM*, *PARP1*	*PARP1*: HOMDEL HETLOSS	*ATM*: HOMDEL HETLOSS	285	17	24	7	2.29	0.003	0.017	Co-occurrence
*RB1*, *BRCA2*	*RB1*: HOMDEL HETLOSS MUT	*BRCA2*: HOMDEL HETLOSS MUT	220	54	21	38	2.882	<0.001	<0.001	Co-occurrence
	*RB1*: HOMDEL HETLOSS MUT	*CHD1*: HOMDEL HETLOSS	218	61	23	31	2.268	<0.001	<0.001	Co-occurrence
	*RB1*: HOMDEL HETLOSS MUT	*SPOP*: MUT = MISSENSE	224	74	17	18	1.68	0.001	0.008	Co-occurrence
*PIK3CA*	*PIK3CA*: AMP GAIN MUT = MISSENSE	*TP53*: HOMDEL HETLOSS MUT	201	27	74	31	1.641	<0.001	<0.001	Co-occurrence
	*PIK3CA*: AMP GAIN MUT = MISSENSE	*AKT1*: AMP GAIN MUT = MISSENSE	270	50	5	8	>3	<0.001	0.002	Co-occurrence
	*PIK3CA*: AMP GAIN MUT = MISSENSE	*MAP3K7*: HOMDEL HETLOSS	188	27	87	31	1.311	0.002	0.009	Co-occurrence
	*PIK3CA*: AMP GAIN MUT = MISSENSE	*SPOP*: MUT = MISSENSE	252	46	23	12	1.515	0.008	0.036	Co-occurrence
	*PIK3CA*: AMP GAIN MUT = MISSENSE	*CHD1*: HOMDEL HETLOSS	237	42	38	16	1.248	0.011	0.045	Co-occurrence
*FOXA1*	*FOXA1*: MUT	*ATM*: HOMDEL HETLOSS	294	8	26	5	2.821	0.004	0.018	Co-occurrence
	*FOXA1*: MUT	*PARP1*: HOMDEL HETLOSS	300	9	20	4	2.737	0.01	0.04	Co-occurrence
	*CHD1*: HOMDEL HETLOSS	*FOXA1*: MUT	274	46	5	8	>3	<0.001	0.002	Co-occurrence
	*MAP3K7*: HOMDEL HETLOSS	*FOXA1*: MUT	211	109	4	9	2.123	0.012	0.047	Co-occurrence
	*RB1*: HOMDEL HETLOSS MUT	*FOXA1*: MUT	237	83	4	9	2.684	0.002	0.01	Co-occurrence
*MYC*	*MYC*: AMP GAIN MUT = MISSENSE	*BRCA1*: HOMDEL HETLOSS MUT	219	76	19	19	1.527	0.002	0.013	Co-occurrence
	*MYC*: AMP GAIN MUT = MISSENSE	*BRCA2*: HOMDEL HETLOSS MUT	212	62	26	33	2.118	<0.001	<0.001	Co-occurrence
	*MYC*: AMP GAIN MUT = MISSENSE	*ATM*: HOMDEL HETLOSS	224	78	14	17	1.802	0.001	0.007	Co-occurrence
	*CHD1*: HOMDEL HETLOSS	*MYC*: AMP GAIN MUT = MISSENSE	209	29	70	25	1.364	0.002	0.01	Co-occurrence
	*MAP3K7*: HOMDEL HETLOSS	*MYC*: AMP GAIN MUT = MISSENSE	169	69	46	49	1.384	<0.001	<0.001	Co-occurrence
	*PIK3CA*: AMP GAIN MUT = MISSENSE	*MYC*: AMP GAIN MUT = MISSENSE	213	25	62	33	2.181	<0.001	<0.001	Co-occurrence
	*RB1*: HOMDEL HETLOSS MUT	*MYC*: AMP GAIN MUT = MISSENSE	185	53	56	39	1.282	<0.001	0.004	Co-occurrence
	*TP53*: HOMDEL HETLOSS MUT	*MYC*: AMP GAIN MUT = MISSENSE	178	60	50	45	1.417	<0.001	<0.001	Co-occurrence

Statistical analysis for trends of co-occurrence and mutual exclusivity between all pairwise comparisons of genomic alterations in the TCGA cohort. Only statistically significant trends are included. Significant genomic subtypes, based on alteration co-occurrences, are highlighted in orange, and alterations that occur across multiple subtypes are shown in gray. Alterations that are mutually exclusive from one another are shown in blue.

## Data Availability

R Code used to generate the multi-cohort oncoprints can be found at: https://github.com/Syksy/curatedPCaData/blob/master/data-raw/oncoprints.R (accessed date: 10 May 2021).

## References

[1] Siegel R.A.-O., Miller K.A.-O., Fuchs H.E., Jemal A. (2020). Cancer Statistics. CA A Cancer J. Clin..

[2] De Marzo A.M., Nelson W.G., Meeker A.K., Coffey D.S. (1998). Stem cell features of benign and malignant prostate epithelial cells. J. Urol..

[3] Cunha G.R., Donjacour A.A., Cooke P.S., Mee S., Bigsby R.M., Higgins S.J., Sugimura Y. (1987). The endocrinology and developmental biology of the prostate. Endocr. Rev..

[4] Buchanan G., Irvine R.A., Coetzee G.A., Tilley W.D. (2001). Contribution of the androgen receptor to prostate cancer predisposition and progression. Cancer Metastasis Rev..

[5] van der Kwast T.H., Schalken J., Ruizeveld de Winter J.A., van Vroonhoven C.C., Mulder E., Boersma W., Trapman J. (1991). Androgen receptors in endocrine-therapy-resistant human prostate cancer. Int. J. Cancer.

[6] Denis L.J., Griffiths K. (2000). Endocrine treatment in prostate cancer. Semin. Surg. Oncol..

[7] Zhu Y., Wen J., Huang G., Mittlesteadt J., Wen X., Lu X.A.-O. (2021). CHD1 and SPOP synergistically protect prostate epithelial cells from DNA damage. Prostate.

[8] Pienta K.J., Bradley D. (2006). Mechanisms underlying the development of androgen-independent prostate cancer. Clin. Cancer Res..

[9] Scher H.I., Fizazi K., Saad F., Taplin M.E., Sternberg C.N., Miller K., de Wit R., Mulders P., Chi K.N., Shore N.D. (2012). Increased survival with enzalutamide in prostate cancer after chemotherapy. N. Engl. J. Med..

[10] Zong Y., Goldstein A.S. (2013). Adaptation or selection--mechanisms of castration-resistant prostate cancer. Nat. Rev. Urol..

[11] Grivas P.D., Robins D.M., Hussain M. (2013). Predicting response to hormonal therapy and survival in men with hormone sensitive metastatic prostate cancer. Crit. Rev. Oncol. Hematol..

[12] Kohli M., Oberg A.L., Mahoney D.W., Riska S.M., Sherwood R., Zhang Y., Zenka R.M., Sahasrabudhe D., Qin R., Zhang S. (2019). Serum Proteomics on the Basis of Discovery of Predictive Biomarkers of Response to Androgen Deprivation Therapy in Advanced Prostate Cancer. Clin. Genitourin. Cancer.

[13] Robinson D., Van Allen E.M., Wu Y.M., Schultz N., Lonigro R.J., Mosquera J.M., Montgomery B., Taplin M.E., Pritchard C.C., Attard G. (2015). Integrative clinical genomics of advanced prostate cancer. Cancer Cell.

[14] Baca S.C., Prandi D., Lawrence M.S., Mosquera J.M., Romanel A., Drier Y., Park K., Kitabayashi N., MacDonald T.Y., Ghandi M. (2013). Punctuated evolution of prostate cancer genomes. Cancer Cell.

[15] Taylor B.S., Schultz N., Hieronymus H., Gopalan A., Xiao Y., Carver B.S., Arora V.K., Kaushik P., Cerami E., Reva B. (2010). Integrative genomic profiling of human prostate cancer. Cancer Cell.

[16] Berger M.F., Lawrence M.S., Demichelis F., Drier Y., Cibulskis K., Sivachenko A.Y., Sboner A., Esgueva R., Pflueger D., Sougnez C. (2011). The genomic complexity of primary human prostate cancer. Nature.

[17] Grasso C.S., Wu Y.M., Robinson D.R., Cao X., Dhanasekaran S.M., Khan A.P., Quist M.J., Jing X., Lonigro R.J., Brenner J.C. (2012). The mutational landscape of lethal castration-resistant prostate cancer. Nature.

[18] Claessens F., Helsen C., Prekovic S., Van den Broeck T., Spans L., Van Poppel H., Joniau S. (2014). Emerging mechanisms of enzalutamide resistance in prostate cancer. Nat. Rev. Urol..

[19] Waltering K.K., Urbanucci A., Visakorpi T. (2012). Androgen receptor (AR) aberrations in castration-resistant prostate cancer. Mol. Cell Endocrinol..

[20] Bubendorf L., Kononen J., Koivisto P., Schraml P., Moch H., Gasser T.C., Willi N., Mihatsch M.J., Sauter G., Kallioniemi O.P. (1999). Survey of gene amplifications during prostate cancer progression by high-throughout fluorescence in situ hybridization on tissue microarrays. Cancer Res..

[21] Abeshouse A., Ahn J., Akbani R., Ally A., Amin S., Andry C.D., Annala M., Aprikian A., Armenia J., Arora A. (2015). The Molecular Taxonomy of Primary Prostate Cancer. Cell.

[22] Barbieri C.E., Baca S.C., Lawrence M.S., Demichelis F., Blattner M., Theurillat J.P., White T.A., Stojanov P., Van Allen E., Stransky N. (2012). Exome sequencing identifies recurrent SPOP, FOXA1 and MED12 mutations in prostate cancer. Nat. Genet..

[23] Hieronymus H., Lamb J., Ross K.N., Peng X.P., Clement C., Rodina A., Nieto M., Du J., Stegmaier K., Raj S.M. (2006). Gene expression signature-based chemical genomic prediction identifies a novel class of HSP90 pathway modulators. Cancer Cell.

[24] Tomlins S.A., Rhodes D.R., Perner S., Dhanasekaran S.M., Mehra R., Sun X.W., Varambally S., Cao X., Tchinda J., Kuefer R. (2005). Recurrent fusion of TMPRSS2 and ETS transcription factor genes in prostate cancer. Science.

[25] Tomlins S.A., Mehra R., Rhodes D.R., Smith L.R., Roulston D., Helgeson B.E., Cao X., Wei J.T., Rubin M.A., Shah R.B. (2006). TMPRSS2:ETV4 gene fusions define a third molecular subtype of prostate cancer. Cancer Res..

[26] Helgeson B.E., Tomlins S.A., Shah N., Laxman B., Cao Q., Prensner J.R., Cao X., Singla N., Montie J.E., Varambally S. (2008). Characterization of TMPRSS2:ETV5 and SLC45A3:ETV5 gene fusions in prostate cancer. Cancer Res..

[27] Perner S., Demichelis F., Beroukhim R., Schmidt F.H., Mosquera J.-M., Setlur S., Tchinda J., Tomlins S.A., Hofer M.D., Pienta K.G. (2006). TMPRSS2:ERG fusion-associated deletions provide insight into the heterogeneity of prostate cancer. Cancer Res..

[28] Tomlins S.A., Laxman B., Dhanasekaran S.M., Helgeson B.E., Cao X., Morris D.S., Menon A., Jing X., Cao Q., Han B. (2007). Distinct classes of chromosomal rearrangements create oncogenic ETS gene fusions in prostate cancer. Nature.

[29] Tomlins S.A., Laxman B., Varambally S., Cao X., Yu J., Helgeson B.E., Cao Q., Prensner J.R., Rubin M.A., Shah R.B. (2008). Role of the TMPRSS2-ERG gene fusion in prostate cancer. Neoplasia.

[30] Bostwick D.G., Liu L., Brawer M.K., Qian J. (2004). High-grade prostatic intraepithelial neoplasia. Rev. Urol..

[31] Lin C., Yang L., Tanasa B., Hutt K., Ju B.-G., Ohgi K., Zhang J., Rose D.W., Fu X.-D., Glass C.K. (2009). Nuclear receptor-induced chromosomal proximity and DNA breaks underlie specific translocations in cancer. Cell.

[32] Haffner M.C., Aryee M.J., Toubaji A., Esopi D.M., Albadine R., Gurel B., Isaacs W.B., Bova G.S., Liu W., Xu J. (2010). Androgen-induced TOP2B-mediated double-strand breaks and prostate cancer gene rearrangements. Nat. Genet..

[33] Mani R.S., Tomlins S.A., Callahan K., Ghosh A., Nyati M.K., Varambally S., Palanisamy N., Chinnaiyan A.M. (2009). Induced chromosomal proximity and gene fusions in prostate cancer. Science.

[34] Sun C., Dobi A., Mohamed A., Li H., Thangapazham R.L., Furusato B., Shaheduzzaman S., Tan S.H., Vaidyanathan G., Whitman E. (2008). TMPRSS2-ERG fusion, a common genomic alteration in prostate cancer activates C-MYC and abrogates prostate epithelial differentiation. Oncogene.

[35] Wright M.E., Tsai M.J., Aebersold R. (2003). Androgen receptor represses the neuroendocrine transdifferentiation process in prostate cancer cells. Mol. Endocrinol..

[36] Yu J., Mani R.-S., Cao Q., Brenner C.J., Cao X., Wang X., Wu L., Li J., Hu M., Gong Y. (2010). An integrated network of androgen receptor, polycomb, and TMPRSS2-ERG gene fusions in prostate cancer progression. Cancer Cell.

[37] Kunderfranco P., Mello-Grand M., Cangemi R., Pellini S., Mensah A., Albertini V., Malek A., Chiorino G., Catapano C.V., Carbone G.M. (2010). ETS transcription factors control transcription of EZH2 and epigenetic silencing of the tumor suppressor gene Nkx3.1 in prostate cancer. PLoS ONE.

[38] Yang L., Mei Q., Zielinska-Kwiatkowska A., Matsui Y., Blackburn M.L., Benedetti D., Krumm A.A., Taborsky G.J., Chansky H.A. (2003). An ERG (ets-related gene)-associated histone methyltransferase interacts with histone deacetylases 1/2 and transcription co-repressors mSin3A/B. Biochem. J..

[39] Graff R.E., Pettersson A., Lis R.T., DuPre N., Jordahl K.M., Nuttall E., Rider J.R., Fiorentino M., Sesso H.D., Kenfield S.A. (2015). The TMPRSS2:ERG fusion and response to androgen deprivation therapy for prostate cancer. Prostate.

[40] Bernasocchi T., El Tekle G.A.-O., Bolis M., Mutti A., Vallerga A., Brandt L.P., Spriano F., Svinkina T., Zoma M., Ceserani V. (2021). Dual functions of SPOP and ERG dictate androgen therapy responses in prostate cancer. Nat. Commun..

[41] Wu M., Shi L., Cimic A., Romero L., Sui G., Lees C.J., Cline J.M., Seals D.F., Sirintrapun J.S., McCoy T.P. (2012). Suppression of Tak1 promotes prostate tumorigenesis. Cancer Res..

[42] Rodrigues L.U., Rider L., Nieto C., Romero L., Karimpour-Fard A., Loda M., Lucia M.S., Wu M., Shi L., Cimic A. (2015). Coordinate loss of MAP3K7 and CHD1 promotes aggressive prostate cancer. Cancer Res..

[43] Liu W., Chang B.L., Cramer S., Koty P.P., Li T., Sun J., Turner A.R., Von Kap-Herr C., Bobby P., Rao J. (2007). Deletion of a small consensus region at 6q15, including the MAP3K7 gene, is significantly associated with high-grade prostate cancers. Clin. Cancer Res..

[44] Kluth M., Hesse J., Heinl A., Krohn A., Steurer S., Sirma H., Simon R., Mayer P.-S., Schumacher U., Grupp K. (2013). Genomic deletion of MAP3K7 at 6q12-22 is associated with early PSA recurrence in prostate cancer and absence of TMPRSS2:ERG fusions. Mod. Pathol..

[45] Jillson L.K., Rider L.C., Rodrigues L.U., Romero L., Karimpour-Fard A., Nieto C., Gillette C.A.-O., Torkko K., Danis E., Smith E.E. (2021). MAP3K7 Loss Drives Enhanced Androgen Signaling and Independently Confers Risk of Recurrence in Prostate Cancer with Joint Loss of CHD1. Mol. Cancer Res..

[46] Ormond D.A.-O., Kleinschmidt-DeMasters B.K., Cavalcante D., Smith E.E., Cramer S.D., Lucia M.S. (2019). Prostatic adenocarcinoma CNS parenchymal and dural metastases: Alterations in ERG, CHD1 and MAP3K7 expression. J. Neurooncol..

[47] Koryakina Y., Ta H.Q., Gioeli D. (2014). Androgen receptor phosphorylation: Biological context and functional consequences. Endocr. Relat. Cancer.

[48] Gioeli D., Black B.E., Gordon V., Spencer A., Kesler C.T., Eblen S.T., Paschal B.M., Weber M.J. (2006). Stress kinase signaling regulates androgen receptor phosphorylation, transcription, and localization. Mol. Endocrinol..

[49] Washino S., Rider L.C., Romero L., Jillson L.K., Affandi T.A.-O., Ohm A.A.-O., Lam E.T., Reyland M.E., Costello J.A.-O., Cramer S.D. (2019). Loss of MAP3K7 Sensitizes Prostate Cancer Cells to CDK1/2 Inhibition and DNA Damage by Disrupting Homologous Recombination. Mol. Cancer Res..

[50] Simic R., Lindstrom D.L., Tran H.G., Roinick K.L., Costa P.J., Johnson A.D., Hartzog G.A., Arndt K.M. (2003). Chromatin remodeling protein Chd1 interacts with transcription elongation factors and localizes to transcribed genes. EMBO J..

[51] Lusser A., Urwin D.L., Kadonaga J.T. (2005). Distinct activities of CHD1 and ACF in ATP-dependent chromatin assembly. Nat. Struct. Mol. Biol..

[52] Augello M.A., Liu D., Deonarine L.D., Robinson B.D., Huang D., Stelloo S., Blattner M., Doane A.S., Wong E.W.P., Chen Y. (2019). CHD1 Loss Alters AR Binding at Lineage-Specific Enhancers and Modulates Distinct Transcriptional Programs to Drive Prostate Tumorigenesis. Cancer Cell.

[53] Zhang Z., Zhou C., Li X., Barnes S.D., Deng S., Hoover E., Chen C.C., Lee Y.S., Zhang Y., Wang C. (2020). Loss of CHD1 Promotes Heterogeneous Mechanisms of Resistance to AR-Targeted Therapy via Chromatin Dysregulation. Cancer Cell.

[54] Kari V., Mansour W.Y., Raul S.K., Baumgart S.J., Mund A., Grade M., Sirma H., Simon R., Will H., Dobbelstein M. (2018). Loss of CHD1 causes DNA repair defects and enhances prostate cancer therapeutic responsiveness. EMBO Rep..

[55] Shenoy T.R., Boysen G., Wang M.Y., Xu Q.Z., Guo W., Koh F.M., Wang C., Zhang L.Z., Wang Y., Gil V. (2017). CHD1 loss sensitizes prostate cancer to DNA damaging therapy by promoting error-prone double-strand break repair. Ann. Oncol..

[56] Mani R.S. (2014). The emerging role of speckle-type POZ protein (SPOP) in cancer development. Drug Discov. Today.

[57] Zhuang M., Calabrese M.F., Liu J., Waddell M.B., Nourse A., Hammel M., Miller D.J., Walden H., Duda D.M., Seyedin S.N. (2009). Structures of SPOP-substrate complexes: Insights into molecular architectures of BTB-Cul3 ubiquitin ligases. Mol. Cell.

[58] Theurillat J.P., Udeshi N.D., Errington W.J., Svinkina T., Baca S.C., Pop M., Wild P.J., Blattner M., Groner A.C., Rubin M.A. (2014). Prostate cancer. Ubiquitylome analysis identifies dysregulation of effector substrates in SPOP-mutant prostate cancer. Science.

[59] Boysen G., Barbieri C.E., Prandi D., Blattner M., Chae S.S., Dahija A., Nataraj S., Huang D., Marotz C., Xu L. (2015). SPOP mutation leads to genomic instability in prostate cancer. Elife.

[60] Blattner M., Liu D., Robinson B.D., Huang D., Poliakov A., Gao D., Nataraj S., Deonarine L.D., Augello M.A., Sailer V. (2017). SPOP Mutation Drives Prostate Tumorigenesis In Vivo through Coordinate Regulation of PI3K/mTOR and AR Signaling. Cancer Cell.

[61] Geng C., He B., Xu L., Barbieri C.E., Eedunuri V.K., Chew S.A., Zimmermann M., Bond R., Shou J., Li C. (2013). Prostate cancer-associated mutations in speckle-type POZ protein (SPOP) regulate steroid receptor coactivator 3 protein turnover. Proc. Natl. Acad. Sci. USA.

[62] An J., Wang C., Deng Y., Yu L., Huang H. (2014). Destruction of full-length androgen receptor by wild-type SPOP, but not prostate-cancer-associated mutants. Cell Rep..

[63] Lai J., Batra J. (2014). Speckle-type POZ protein mutations interrupt tumor suppressor function of speckle-type POZ protein in prostate cancer by affecting androgen receptor degradation. Asian J. Androl..

[64] Geng C., Rajapakshe K., Shah S.S., Shou J., Eedunuri V.K., Foley C., Fiskus W., Rajendran M., Chew S.A., Zimmermann M. (2019). Androgen receptor is the key transcriptional mediator of the tumor suppressor SPOP in prostate cancer. Cancer Res..

[65] Yan Y., An J., Yang Y., Wu D., Bai Y., Cao W., Ma L., Chen J., Yu Z., He Y. (2018). Dual inhibition of AKT-mTOR and AR signaling by targeting HDAC3 in PTEN- or SPOP-mutated prostate cancer. EMBO Mol. Med..

[66] Dai X., Gan W., Li X., Wang S., Zhang W., Huang L., Liu S., Zhong Q.A.-O.X., Guo J., Zhang J. (2017). Prostate cancer-associated SPOP mutations confer resistance to BET inhibitors through stabilization of BRD4. Nat. Med..

[67] Maehama T., Dixon J.E. (1998). The tumor suppressor, PTEN/MMAC1, dephosphorylates the lipid second messenger, phosphatidylinositol 3,4,5-trisphosphate. J. Biol. Chem..

[68] Saal L.H., Johansson P., Holm K., Gruvberger-Saal S.K., She Q.-B., Maurer M., Koujak S., Ferrando A., Malmström P., Memeo L. (2007). Poor prognosis in carcinoma is associated with a gene expression signature of aberrant PTEN tumor suppressor pathway activity. Proc. Natl. Acad. Sci. USA.

[69] Krohn A., Diedler T., Burkhardt L., Mayer P.-S., De Silva C., Meyer-Kornblum M., Kötschau D., Tennstedt P., Huang J., Gerhäuser C. (2012). Genomic deletion of PTEN is associated with tumor progression and early PSA recurrence in ERG fusion-positive and fusion-negative prostate cancer. Am. J. Pathol..

[70] Kaarbø M., Mikkelsen O.L., Malerød L., Qu S., Lobert V.H., Akgul G., Halvorsen T., Maelandsmo G.M., Saatcioglu F. (2010). PI3K-AKT-mTOR pathway is dominant over androgen receptor signaling in prostate cancer cells. Cell Oncol..

[71] Carver B.S., Chapinski C., Wongvipat J., Hieronymus H., Chen Y., Chandarlapaty S., Arora V.K., Le C., Koutcher J., Scher H. (2011). Reciprocal feedback regulation of PI3K and androgen receptor signaling in PTEN-deficient prostate cancer. Cancer Cell.

[72] Mulholland D.J., Tran L.M., Li Y., Cai H., Morim A., Wang S., Plaisier S., Garraway I.P., Huang J., Graeber T.G. (2011). Cell autonomous role of PTEN in regulating castration-resistant prostate cancer growth. Cancer Cell.

[73] Lee S.H., Johnson D., Luong R., Sun Z. (2015). Crosstalking between androgen and PI3K/AKT signaling pathways in prostate cancer cells. J. Biol. Chem..

[74] Lin H.K., Yeh S., Kang H.Y., Chang C. (2001). Akt suppresses androgen-induced apoptosis by phosphorylating and inhibiting androgen receptor. Proc. Natl. Acad. Sci. USA.

[75] Schwartz S., Wongvipat J., Trigwell C.B., Hancox U., Carver B.S., Rodrik-Outmezguine V., Will M., Yellen P., de Stanchina E., Baselga J. (2015). Feedback suppression of PI3Kα signaling in PTEN-mutated tumors is relieved by selective inhibition of PI3Kβ. Cancer Cell.

[76] Klempner S.J., Myers A.P., Cantley L.C. (2013). What a tangled web we weave: Emerging resistance mechanisms to inhibition of the phosphoinositide 3-kinase pathway. Cancer Discov..

[77] Chen Z., Trotman L.C., Shaffer D., Lin H.-K., Dotan Z.A., Niki M., Koutcher J.A., Scher H.I., Ludwig T., Gerald W. (2005). Crucial role of p53-dependent cellular senescence in suppression of Pten-deficient tumorigenesis. Nature.

[78] Zhou Z., Flesken-Nikitin A., Corney D.C., Wang W., Goodrich D.W., Roy-Burman P., Nikitin A.Y. (2006). Synergy of p53 and Rb deficiency in a conditional mouse model for metastatic prostate cancer. Cancer Res..

[79] Burchardt M., Burchardt T., Shabsigh A., Ghafar M., Chen M.W., Anastasiadis A., de la Taille A., Kiss A., Buttyan R. (2001). Reduction of wild type p53 function confers a hormone resistant phenotype on LNCaP prostate cancer cells. Prostate.

[80] Navone N.M., Troncoso P., Pisters L.L., Goodrow T.L., Palmer J.L., Nichols W.W., von Eschenbach A.C., Conti C.J. (1993). p53 protein accumulation and gene mutation in the progression of human prostate carcinoma. J. Natl. Cancer Inst..

[81] Cronauer M.V., Troncoso P., Pisters L.L., Goodrow T.L., Palmer J.L., Nichols W.W., von Eschenbach A.C., Conti. C.J. (2004). Inhibition of p53 function diminishes androgen receptor-mediated signaling in prostate cancer cell lines. Oncogene.

[82] Chappell W.H., Lehmann B.D., Terrian D.M., Abrams S.L., Steelman L.S., McCubrey J.A. (2012). p53 expression controls prostate cancer sensitivity to chemotherapy and the MDM2 inhibitor Nutlin-3. Cell Cycle.

[83] Maddison L.A., Sutherland B.W., Barrios R.J., Greenberg N.M. (2004). Conditional deletion of Rb causes early stage prostate cancer. Cancer Res..

[84] Sharma A., Yeow W.S., Ertel A., Coleman I., Clegg N., Thangavel C., Morrissey C., Zhang X., Comstock C.E.S., Witkiewicz A.K. (2010). The retinoblastoma tumor suppressor controls androgen signaling and human prostate cancer progression. J. Clin. Investig..

[85] Faisal F.A., Murali S., Kaur H., Vidotto T.A.-O., Guedes L.B., Salles D.C., Kothari V., Tosoian J.A.-O., Han S., Hovelson D.H. (2020). CDKN1B Deletions are Associated with Metastasis in African American Men with Clinically Localized, Surgically Treated Prostate Cancer. Clin. Cancer Res..

[86] Cirillo L.A., Lin F.R., Cuesta I., Friedman D., Jarnik M., Zaret K.S. (2002). Opening of compacted chromatin by early developmental transcription factors HNF3 (FoxA) and GATA-4. Mol. Cell.

[87] Cirillo L.A., McPherson C.E., Bossard P., Stevens K., Cherian S., Shim E.Y., Clark K.L., Burley S.K., Zaret K.S. (1998). Binding of the winged-helix transcription factor HNF3 to a linker histone site on the nucleosome. EMBO J..

[88] Parolia A., Cieslik M., Chu S.C., Xiao L., Ouchi T., Zhang Y., Wang X., Vats P., Cao X., Pitchiaya S. (2019). Distinct structural classes of activating FOXA1 alterations in advanced prostate cancer. Nature.

[89] Xu B., Song B., Lu X., Kim J., Hu M., Zhao J.C., Yu J. (2019). Altered chromatin recruitment by FOXA1 mutations promotes androgen independence and prostate cancer progression. Cell Res..

[90] Gao N., Zhang J., Rao M.A., Case T.C., Mirosevich J., Wang Y., Jin R., Gupta A., Rennie P.S., Matusik R.J. (2003). The role of hepatocyte nuclear factor-3 alpha (Forkhead Box A1) and androgen receptor in transcriptional regulation of prostatic genes. Mol. Endocrinol..

[91] Jin H.J., Zhao J.C., Wu L., Kim J., Yu J. (2014). Cooperativity and equilibrium with FOXA1 define the androgen receptor transcriptional program. Nat. Commun..

[92] Adams E.J., Karthaus W.R., Hoover E., Liu D., Gruet A., Zhang Z., Cho H., DiLoreto R., Chhangawala S., Liu Y. (2019). FOXA1 mutations alter pioneering activity, differentiation and prostate cancer phenotypes. Nature.

[93] Gao S.A.-O., Chen S., Han D., Barrett D., Han W.A.-O., Ahmed M., Patalano S., Macoska J.A., He H.H., Cai C.A.-O. (2019). Forkhead domain mutations in FOXA1 drive prostate cancer progression. Cell Res..

[94] Jain R.K., Mehta R.J., Nakshatri H., Idrees M.T., Badve S.S. (2011). High-level expression of forkhead-box protein A1 in metastatic prostate cancer. Histopathology.

[95] Messina C., Cattrini C., Soldato D., Vallome G., Caffo O., Castro E., Olmos D., Boccardo F., Zanardi E.A.-O. (2020). BRCA Mutations in Prostate Cancer: Prognostic and Predictive Implications. J. Oncol..

[96] Mak J.A.-O., Ma H.T., Poon R.A.-O. (2020). Synergism between ATM and PARP1 Inhibition Involves DNA Damage and Abrogating the G(2) DNA Damage Checkpoint. Mol. Cancer Ther..

[97] Awasthi P., Foiani M., Kumar A. (2015). ATM and ATR signaling at a glance. J. Cell Sci..

[98] Polkinghorn W.R., Parker J.S., Lee M.X., Kass E.M., Spratt D.E., Iaquinta P.J., Arora V.K., Yen W.-F., Cai L., Zheng D. (2013). Androgen receptor signaling regulates DNA repair in prostate cancers. Cancer Discov..

[99] Goodwin J.F., Schiewer M.J., Dean J.L., Schrecengost R.S., de Leeuw R., Han S., Ma T., Den R.B., Dicker A.P., Feng F.Y. (2013). A hormone-DNA repair circuit governs the response to genotoxic insult. Cancer Discov..

[100] Spratt D.E., Evans M.J., Davis B.J., Doran M.G., Lee M.X., Shah N., Wongvipat J., Carnazza K.E., Klee G.G., Polkinghorn W. (2015). Androgen Receptor Upregulation Mediates Radioresistance after Ionizing Radiation. Cancer Res..

[101] Siddiqui Z.A., Krauss D.J. (2018). Adjuvant androgen deprivation therapy for prostate cancer treated with radiation therapy. Transl. Androl. Urol..

[102] Spratt D.E., Malone S., Roy S., Grimes S., Eapen L., Morgan S.C., Malone J., Craig J., Dess R.T., Jackson W.C. (2021). Prostate Radiotherapy with Adjuvant Androgen Deprivation Therapy (ADT) Improves Metastasis-Free Survival Compared to Neoadjuvant ADT: An Individual Patient Meta-Analysis. J. Clin. Oncol..

[103] Jenkins R.B., Qian J., Lieber M.M., Bostwick D.G. (1997). Detection of c-myc oncogene amplification and chromosomal anomalies in metastatic prostatic carcinoma by fluorescence in situ hybridization. Cancer Res..

[104] Fromont G., Godet J., Peyret A., Irani J., Celhay O., Rozet F., Cathelineau X., Cussenot O. (2013). 8q24 amplification is associated with Myc expression and prostate cancer progression and is an independent predictor of recurrence after radical prostatectomy. Hum. Pathol..

[105] Barfeld S.J., Fazli L., Persson M., Marjavaara L., Urbanucci A., Kaukoniemi K.M., Rennie P.S., Ceder Y., Chabes A., Visakorpi T. (2015). Myc-dependent purine biosynthesis affects nucleolar stress and therapy response in prostate cancer. Oncotarget.

[106] Koh C.M., Gurel B., Sutcliffe S., Aryee M.J., Schultz D., Iwata T., Uemura M., Zeller K.I., Anele U., Zheng Q. (2011). Alterations in nucleolar structure and gene expression programs in prostatic neoplasia are driven by the MYC oncogene. Am. J. Pathol..

[107] Ellwood-Yen K., Graeber T.G., Wongvipat J., Iruela-Arispe M.L., Zhang J., Matusik R., Thomas G.V., Sawyers C.L. (2003). Myc-driven murine prostate cancer shares molecular features with human prostate tumors. Cancer Cell..

[108] Iwata T., Schultz D., Hicks J., Hubbard G.K., Mutton L.N., Lotan T.L., Bethel C., Lotz M.T., Yegnasubramanian S., Nelson W.G. (2010). MYC overexpression induces prostatic intraepithelial neoplasia and loss of Nkx3.1 in mouse luminal epithelial cells. PLoS ONE.

[109] Gurel B., Iwata T., Koh C.M., Jenkins R.B., Lan F., Van Dang C., Hicks J.L., Morgan J., Cornish T.C., Sutcliffe S. (2008). Nuclear MYC protein overexpression is an early alteration in human prostate carcinogenesis. Mod. Pathol..

[110] Vander Griend D.J., Litvinov I.V., Isaacs J.T. (2014). Conversion of androgen receptor signaling from a growth suppressor in normal prostate epithelial cells to an oncogene in prostate cancer cells involves a gain of function in c-Myc regulation. Int. J. Biol. Sci..

[111] Barfeld S.J., Urbanucci A., Itkonen H.M., Fazli L., Hicks J.L., Thiede B., Rennie P.S., Yegnasubramanian S., DeMarzo A.M., Mills I.G. (2017). c-Myc Antagonises the Transcriptional Activity of the Androgen Receptor in Prostate Cancer Affecting Key Gene Networks. EBioMedicine.

[112] Bernard D., Pourtier-Manzanedo A., Gil J., Beach D.H. (2003). Myc confers androgen-independent prostate cancer cell growth. J. Clin. Investig..

[113] Bai S., Cao S., Jin L., Kobelski M., Schouest B., Wang X., Ungerleider N., Baddoo M., Zhang W., Corey E. (2019). A positive role of c-Myc in regulating androgen receptor and its splice variants in prostate cancer. Oncogene.

[114] Asangani I.A., Dommeti V.L., Wang X., Malik R., Cieslik M., Yang R., Escara-Wilke J., Wilder-Romans K., Dhanireddy S., Engelke C. (2014). Therapeutic targeting of BET bromodomain proteins in castration-resistant prostate cancer. Nature.

[115] Rebello R.J., Kusnadi E., Cameron D.P., Pearson H.B., Lesmana A., Devlin J.R., Drygin D., Clark A.K., Porter L., Pedersen J. (2016). The Dual Inhibition of RNA Pol I Transcription and PIM Kinase as a New Therapeutic Approach to Treat Advanced Prostate Cancer. Clin. Cancer Res..

[116] Boysen G., Rodrigues D.N., Rescigno P., Seed G., Dolling D., Riisnaes R., Crespo M., Zafeiriou Z., Sumanasuriya S., Bianchini D. (2018). SPOP-Mutated/CHD1-Deleted Lethal Prostate Cancer and Abiraterone Sensitivity. Clin. Cancer Res..

[117] Brenner J.C., Ateeq B., Li Y., Yocum A.K., Cao Q., Asangani I.A., Patel S., Wang X., Liang H., Yu J. (2011). Mechanistic rationale for inhibition of poly(ADP-ribose) polymerase in ETS gene fusion-positive prostate cancer. Cancer Cell.

[118] Schiewer M.J., Goodwin J.F., Han S., Brenner J.C., Augello M.A., Dean J.L., Liu F., Planck J.L., Ravindranathan P., Chinnaiyan A.M. (2012). Dual roles of PARP-1 promote cancer growth and progression. Cancer Discov..

[119] Carver B.S., Tran J., Gopalan A., Chen Z., Shaikh S., Carracedo A., Alimonti A., Nardella C., Varmeh S., Scardino P.T. (2020). Aberrant ERG expression cooperates with loss of PTEN to promote cancer progression in the prostate. Nat. Genet..

[120] King J.C., Xu J., Wongvipat J., Hieronymus H., Carver B.S., Leung D.H., Taylor B.S., Sander C., Cardiff R.D., Couto S.S. (2009). Cooperativity of TMPRSS2-ERG with PI3-kinase pathway activation in prostate oncogenesis. Nat. Genet..

[121] Chen Y., Chi P., Rockowitz S., Iaquinta P.J., Shamu T., Shukla S., Gao D., Sirota I., Carver B.S., Wongvipat J. (2013). ETS factors reprogram the androgen receptor cistrome and prime prostate tumorigenesis in response to PTEN loss. Nat. Med..

[122] Mao N., Gao D., Hu W., Hieronymus H., Wang S., Lee Y.A.-O., Lee C.A.-O., Choi D., Gopalan A., Chen Y. (2019). Aberrant Expression of ERG Promotes Resistance to Combined PI3K and AR Pathway Inhibition through Maintenance of AR Target Genes. Mol. Cancer Ther..

[123] Martin P., Liu Y.N., Pierce R., Abou-Kheir W., Casey O., Seng V., Camacho D., Simpson R.M., Kelly K. (2011). Prostate epithelial Pten/TP53 loss leads to transformation of multipotential progenitors and epithelial to mesenchymal transition. Am. J. Pathol..

[124] Blee A.M., He Y., Yang Y., Ye Z., Yan Y., Pan Y., Ma T., Dugdale J., Kuehn E., Kohli M. (2018). TMPRSS2-ERG Controls Luminal Epithelial Lineage and Antiandrogen Sensitivity in PTEN and TP53-Mutated Prostate Cancer. Clin. Cancer Res..

[125] Mateo J., Carreira S., Sandhu S., Miranda S., Mossop H., Perez-Lopez R., Nava Rodrigues D., Robinson D., Omlin A., Tunariu N. (2015). DNA-Repair Defects and Olaparib in Metastatic Prostate Cancer. N. Engl. J. Med..

[126] Pezaro C. (2020). PARP inhibitor combinations in prostate cancer. Ther. Adv. Med. Oncol..

[127] Luo J., Antonarakis E.S. (2019). PARP inhibition - not all gene mutations are created equal. Nat. Rev. Urol..

[128] Jette N.R., Kumar M., Radhamani S., Arthur G., Goutam S., Yip S., Kolinsky M., Williams G.J., Bose P., Lees-Miller S.P. (2020). ATM-Deficient Cancers Provide New Opportunities for Precision Oncology. Cancers (Basel).

[129] Rafiei S., Fitzpatrick K., Liu D.A.-O., Cai M.Y., Elmarakeby H.A.-O., Park J.A.-O., Ricker C., Kochupurakkal B.S., Choudhury A.A.-O., Hahn W.C. (2020). ATM Loss Confers Greater Sensitivity to ATR Inhibition Than PARP Inhibition in Prostate Cancer. Cancer Res..

[130] Marshall C.H., Sokolova A.O., McNatty A.L., Cheng H.H., Eisenberger M.A., Bryce A.H., Schweizer M.T., Antonarakis E.S. (2019). Differential Response to Olaparib Treatment Among Men with Metastatic Castration-resistant Prostate Cancer Harboring BRCA1 or BRCA2 Versus ATM Mutations. Eur. Urol..

[131] Neeb A., Herranz N., Arce-Gallego S., Miranda S., Buroni L., Yuan W., Athie A., Casals T., Carmichael J., Rodrigues D.N. (2021). Advanced Prostate Cancer with ATM Loss: PARP and ATR Inhibitors. Eur. Urol..

[132] Lloyd R.L., Wijnhoven P.W.G., Ramos-Montoya A., Wilson Z., Illuzzi G., Falenta K., Jones G.N., James N., Chabbert C.D., Stott J. (2020). Combined PARP and ATR inhibition potentiates genome instability and cell death in ATM-deficient cancer cells. Oncogene.

[133] Chakraborty G., Armenia J., Mazzu Y.Z., Nandakumar S., Stopsack K.A.-O., Atiq M.A.-O., Komura K.A.-O., Jehane L., Hirani R., Chadalavada K. (2020). Significance of BRCA2 and RB1 Co-loss in Aggressive Prostate Cancer Progression. Clin. Cancer Res..

[134] Chatterjee P., Schweizer M.T., Lucas J.M., Coleman I., Nyquist M.D., Frank S.B., Tharakan R., Mostaghel E., Luo J., Pritchard C.C. (2019). Supraphysiological androgens suppress prostate cancer growth through androgen receptor-mediated DNA damage. Clin. Investig..

[135] Mohammad O.S., Nyquist M.D., Schweizer M.T., Balk S.P., Corey E., Plymate S., Nelson P.S., Mostaghel E.A. (2017). Supraphysiologic Testosterone Therapy in the Treatment of Prostate Cancer: Models, Mechanisms and Questions. Cancers.

[136] Nyquist M.D., Ang L.S., Corella A., Coleman I.M., Meers M.P., Christiani A.J., Pierce C., Janssens D.H., Meade H.E., Bose A. (2021). Selective androgen receptor modulators activate the canonical prostate cancer androgen receptor program and repress cancer growth. J. Clin. Investig..

